# Pharmacokinetic variability of beta‐adrenergic blocking agents used in cardiology

**DOI:** 10.1002/prp2.496

**Published:** 2019-07-12

**Authors:** Frederik N. Ågesen, Peter E. Weeke, Peer Tfelt‐Hansen, Jacob Tfelt‐Hansen, for ESCAPE‐NET

**Affiliations:** ^1^ Department of Cardiology University of Copenhagen Rigshospitalet, Copenhagen Denmark; ^2^ Danish Headache Center, Department of Neurology University of Copenhagen, Rigshospitalet‐Glostrup Hospital Glostrup Denmark

**Keywords:** beta‐adrenergic blocking agents, metoprolol, personalized medicine, Pharmacokinetics, pharmacology, propranolol

## Abstract

The aim of this study was to evaluate the pharmacokinetic variability of beta‐adrenergic blocking agents used in cardiology by reviewing single‐dose and steady‐state pharmacokinetic studies from the literature. PubMed was searched for pharmacokinetic studies of beta‐adrenergic blocking agents, both single‐dose and steady‐state studies. The studies included reported maximum plasma concentration (C_max_) and/or area under the concentration curve (AUC). The coefficient of variation (CV%) was calculated for all studies, and a CV% <40% was considered low or moderate variability, and a CV% >40% was considered high variability. The C_max_ and AUC were reported a total of 672 times in 192 papers. Based on AUC, metoprolol, propranolol, carvedilol, and nebivolol showed high pharmacokinetic variability (highest first), whereas bisoprolol, atenolol, sotalol, labetalol, nadolol, and pindolol showed low to moderate variability (lowest first). We have shown a high interindividual pharmacokinetic variability that varies markedly in different beta‐adrenergic blocking agents; the extreme being steady state ratios as high as 30 in metoprolol. A more personalized approach to the medical treatment of patients may be obtained by combining known pharmacokinetic information about variability, pharmaco‐genetics and ‐dynamics, and patient characteristics, to avoid adverse events or lack of treatment effect.

**Abbreviations**
ADME: absorption, distribution, metabolism, and excretion
AUC: area under the concentration curve
CI: confidence interval
Cmax: maximum plasma concentration
CV%: coefficient of variation
CYP2D6: Cytochrome p450 2D6
RCTs: randomized controlled trials
SS: steady‐state studies

## 1 INTRODUCTION

Beta‐adrenergic blocking agents (β‐blockers) are part of the standard care in prevention or treatment of cardiovascular disease such as myocardial infarction, heart failure, arrhythmias, and hypertension, making β‐blockers some of the most widely used prescription drugs worldwide.^1^ β‐blockers have shown significantly reduced mortality rates in heart failure patients and is the cornerstone in prevention of sudden cardiac death in long QT syndrome (LQTS) patients.^2^, ^3^ β‐blockers have thus in heart failure shown “marked beneficial effects, on cardiac function, morbidity, and survival”.^4^ The clinical effects are, however, affected by significant intraclass and interpatient variability.^4^

It is important to distinguish between different attributes (intraclass variability) among β‐blockers and the interpatient variability in clinical responses. The clinical response depends on a multitude of varying factors: pharmacokinetic variability of the drug, variability of the pharmacodynamics, eg dose–response curves, variability of the disease treated, drug–drug interactions, and presence of comorbidity. Therefore, clinicians cannot assume a “class effect” with all β‐blockers.^5^

The subject of the present review is the interindividual pharmacokinetic variability for each of the β‐blockers with a proven efficacy in cardiology, with an emphasis on aspects relevant for the therapeutics of these drugs.

The influence of the pharmacokinetic factors absorption, distribution, metabolism, and excretion (ADME) are of relevance when a varied drug response in patients is observed.^6^ Interindividual differences such as permeability in the intestines, binding to serum proteins, first–pass metabolism, and degree of renal insufficiency with regards to hydrophilic β‐blockers are important regarding pharmacokinetics. Cytochrome p450 2D6 (CYP2D6) gene expression is an example of this, as it induces increased exposure and risk of adverse events in poor metabolizers of metoprolol, while, on the other hand, a regular dose of metoprolol administered to an ultrarapid metabolizer might prove ineffective.^7^, ^8^

Ultimately, an untargeted β‐blocker dosing has retrospectively shown to significantly increase mortality in patients with chronic systolic heart failure patients compared to target dose or target heart rhythm, why a more targeted approach may improve the outcome when initiating β‐blocker treatment.^9^

Numerous clinical studies on the pharmacokinetics of β‐blockers have been conducted since the 1960’s.^10^, ^11^ The purpose of this study is to review and analyze the pharmacokinetic variability of β‐blockers in healthy individuals who received single or multiple doses in pharmacokinetic studies to provide some guidance for safer and personalized use of β‐blockers in the clinic and diminish adverse events or ineffectiveness of treatment in patients.

## 2 METHODS

We searched PubMed until June 2018 for studies on pharmacokinetics of 14 β‐blockers used in clinical cardiology and which have demonstrated superiority over placebos in at least two randomized controlled trials (RCTs): Atenolol, betaxolol, bisoprolol, carvedilol, esmolol, labetalol, metoprolol, nadolol, nebivolol, oxprenolol, pindolol, propranolol, sotalol, and timolol.

PubMed was searched for each drug with the terms “generic drug name” and “pharmacokinetics” for both single‐dose and steady‐state studies: I “generic name AND pharmacokinetics AND single dose” for single‐dose studies; II “generic name AND pharmacokinetics AND (steady state OR multiple dose)” for steady‐state studies.

The pharmacokinetic results in oral single‐dose or steady‐state studies in healthy adult volunteers were included for analysis of variability. Persons with arterial hypertension, but with normal hepatic and renal function, were included. Studies with a β‐blocker and an oral placebo administered simultaneously were included, while studies with simultaneous intake of other medication were excluded. Studies presenting only results as means or individual data in figures were excluded from this study, since no coefficient of variation (CV) could be calculated.

The pharmacokinetic variability was expressed as the CV for the maximum plasma concentration (C_max_) and the area under the concentration curve (AUC). For C_max_ and AUC, we classified the CV of each study (single‐dose or steady‐state) as: <20%, 20%‐40%, 40%‐60%, 60%‐80%, or > 80%.

The variability of the pharmacokinetic parameters was most often presented in the individual studies as mean and standard deviation (SD) or as mean and standard error of the mean (SEM). SD was used to calculate the CV: CV = SD/mean. SEM was calculated back to SD with the formula: SD = SEM $\sqrt{n}$ with *n* being the number of subjects studied, and the calculated SD was then used in calculating CV.

The pharmacokinetic variability of a drug for each study was divided into two categories: high (CV > 40%) and low or moderate (CV < 40%) variability as proposed by Rowland and Tozer.^12^

When available, the ratio of maximum to minimum value of AUC between participants was presented or could be obtained from the presented raw data, with the AUC ratio being the maximum AUC value divided by the minimum AUC value.

## 3 RESULTS

Overall, we included a total of 192 publications investigating the pharmacokinetics of oral β‐blockers among healthy individuals and persons with hypertension. Thirteen β‐blockers were included, see Table 1. While we initially identified 14 different β‐blockers that are commonly used for treatment of cardiovascular disease, we were only able to identify studies on 13 of these, as no pharmacokinetic studies on esmolol were identified. No steady‐state pharmacokinetic studies were found for timolol.

**Table 1:** Pharmacokinetic variability in single‐dose (SD) and steady‐state (SS) studies of 13 beta‐adrenergic blocking agents

Drug	Study type	CV parameter	Doses (mg)	CV 0%‐20%	CV 20%‐40%	CV 40%‐60%	CV 60%‐80%	CV > 80%	References (Supplementary Materials)
Atenolol	SD	C_max_	25‐200	5	20	10	1		^35‐56^
		AUC	25‐200	8	23	4		1	^35‐56^
	SS	C_max_	25‐100	1	4	2			^37,57‐60^
		AUC	50‐100	1	3				^37,58‐60^
Betaxolol	SD	C_max_	20		1				^61^
		AUC	20		1				^61^
	SS	C_max_	20	1					^62^
		AUC	20		1				^62^
Bisoprolol	SD	C_max_	5‐100	10	2				^63‐70^
		AUC	5‐40	7	3				^64‐69^
	SS	C_max_	10		2				^71,72^
		AUC	10		2				^71,72^
Carvedilol	SD	C_max_	6,25‐128	1		5	6	1	^73‐81^
		AUC	6,25‐128	2		4	6		^73‐76,78‐81^
	SS	C_max_	25			1			^73^
		AUC	25			1			^73^
Labetalol	SD	C_max_	100‐400	4	2	8	2		^55,82‐86^
		AUC	100‐200	3	6	4	1		^55,82,84‐86^
	SS	C_max_	400		2				^87^
		AUC	100‐400		3		1		^87‐89^
Metoprolol	SD	C_max_	25‐200	1	9	24	6	5	^38,65,90‐118^
		AUC	25‐200	1	7	18	11	12	^38,65,91‐123^
	SS	C_max_	50‐400	2	13	19	9	6	^17,58,124‐141^
		AUC	50‐400	3	5	12	6	14	^17,58,119,126‐131,133‐141^
Nadolol	SD	C_max_	30‐120		2	3			^142‐144^
		AUC	30‐80		5	1			^142‐145^
	SS	C_max_	10‐80	2	1	2			^143,146^
		AUC	10‐80		3	3			^143,145,146^
Nebivolol	SD	C_max_	5‐10	1	1	8	1		^147‐154^
		AUC	5‐10	1	2		2	6	^147‐154^
	SS	C_max_	20			1			^155^
		AUC	20				1		^155^
Oxprenolol	SD	C_max_	80‐260	2	1				^156,157^
		AUC	80‐260	2	1	1			^122,156,157^
	SS	C_max_	160		1	2			^126,158^
		AUC	160		1	2			^126,158^
Pindolol	SD	C_max_	5‐30	4	5	1		2	^159‐165^
		AUC	5‐30	4	4	1		2	^159‐165^
	SS	C_max_	1‐5		2	4			^166,167^
		AUC	1‐15		3	3	1		^166‐168^
Propranolol	SD	C_max_	0,5‐320	3	8	29	7	4	^38,42,51,70,93,95,169‐191^
		AUC	0,5‐320	3	15	23	8	9	^38,42,51,93,95,169‐186,188‐198^
	SS	C_max_	40‐180		5	11	6		^59,62,128,171,180,188,199‐209^
		AUC	40‐180		3	23	8	5	^59,62,128,166,171,180,188,195,197,199‐214^
Sotalol	SD	C_max_	40‐320	9	4	1			^215‐220^
		AUC	40‐320	10	4				^215‐220^
	SS	C_max_	160‐320	1				1	^218,221^
		AUC	80‐400	4		1			^217,218,221,222^
Timolol	SD	C_max_	10‐120	1	3	1	1		^223‐225^
		AUC	0,4‐60		2	2	3		^142,193,223‐225^

C_max_ maximum serum concentration, AUC area under the concentration‐time curve.

The C_max_ was reported in 225 single‐dose and 101 steady‐state pharmacokinetic studies of β‐blockers, and the AUC was reported in 233 single‐dose studies and 113 steady‐state pharmacokinetic studies. Table 1 shows the distribution of numbers of studies with CV < 20%, 20%‐40%, 40%‐60%, 60%‐80%, or > 80% for C_max_ and AUC in single‐dose and steady‐state pharmacokinetic studies of the 13 β‐blockers.

The distribution of CVs for AUCs in single‐dose and steady‐state studies being < 40% (low or moderate variability) or > 40% (high variability) is shown in Table 2. The number of studies for each β‐blocker ranged from two studies to 111 studies. Propranolol and metoprolol show a somewhat identical number of studies with a CV of AUC > 40% in both single‐dose and steady–state studies (69% and 92% vs. 84% and 80%, respectively).

**Table 2:** Distribution of coefficients of variance (CV) for area under the plasma concentration‐time curve (AUC) in pharmacokinetic studies of beta‐adrenergic blocking agents where the CVs were > 40% (high variability)

Drug	Number (%) of single‐dose studies with CV > 40% for AUC	Number (%) of steady‐state studies with CV > 40% for AUC	References (Supplementary Materials)
Atenolol	5/36 (14)	0/4 (0)	^41,42,51,54^
Betaxolol	0/1 (0)	0/1 (0)	
Bisoprolol	0/10 (0)	0/2 (0)	
Carvedilol	10/12 (83)	1/1 (100)	^73,74,76,78,80,81^
Labetalol	5/14 (36)	1/4 (25)	^55,86,89^
Metoprolol	41/49 (84)	32/40 (80)	^17,38,58,65,91‐98,100‐107,109‐115,117‐123,126‐131,135‐141^
Nadolol	1/6 (17)	3/6 (50)	^145,146^
Nebivolol	8/11 (73)	1/1 (100)	^148‐150,152‐155^
Oxprenolol	1/4 (25)	2/3 (66)	^122,126,158^
Pindolol	3/11 (27)	4/7 (57)	^163,165‐167^
Propranolol	40/58 (69)	36/39 (92)	^38,42,51,59,62,93,95,128,166,170‐172,175‐178,180‐182,184‐186,188‐190,194‐205,207‐214^
Sotalol	0/14 (0)	1/5 (20)	^222^
Timolol	5/7 (71)	ND	^193,223‐225^

ND, no data.

**Figure 1:**
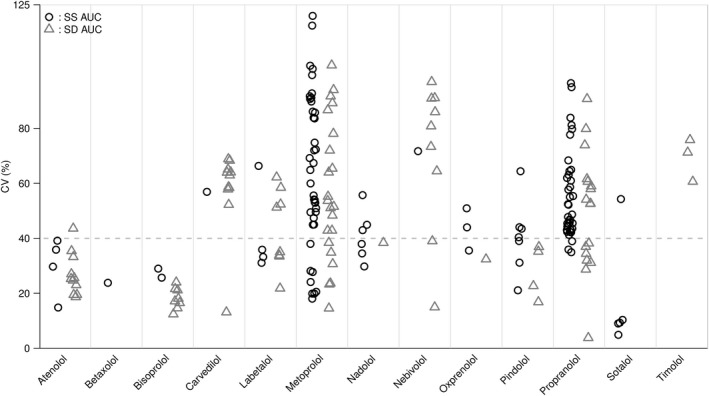
Cloud plot of the distribution of the coefficient of variance (CV) in beta‐adrenergic blocking agents for the area under the plasma‐concentration time curve (AUC) in steady‐state studies (SS) and in single‐dose (SD) studies with AUC extrapolated to infinity

The variability of the CV% for AUC in steady‐state studies with fixed time points as well as in single‐dose studies with time from zero calculated to infinity using the trapezoid rule for AUC is illustrated in Figure 1.

In the steady‐state studies 95% of studies were conducted in Europe or North America and 5% in Asia. The median lower age limit of participants was 21 years (interquartile range, IQR, 20‐23) and median upper age limit 37 (IQR 32‐45). Approximately 75% of studies specified the sex of participants. The sex ratio was 8:1 (males to females).

In single‐dose studies 70% of studies were conducted in Europe or North America, 29% in Asia, and 1% in South America, the median lower age limit of participants was 21 years (IQR 19.5‐23) and median upper age limit 42 (IQR 34‐55). The sex ratio was 4:1.

An overview of the pharmacokinetic properties of the studied β‐blockers is shown in Table 3.

**Table 3:** Pharmacokinetics properties of beta‐adrenergic blocking agents (with permission from Frishman^22^ and publisher)

Drug	Extent of absorption (% of dose)	Bioavailability (% of dose)	Major first–pass hepatic metabolism	Lipid solubility
Atenolol	≈50	≈40	No	Low
Betaxolol	>90	≈80	No	Low
Bisoprolol	≈90	≈88	No	Low
Carvedilol	>90	≈30	Yes	Moderate
Labetalol	>90	≈33	Yes	Moderate
Metoprolol	>90	≈50	Yes	Moderate
Nadolol	≈30	≈30	No	Low
Nebivolol	>90	12‐96	Yes	Low^a^
Oxprenolol	≈90	19‐74	Yes	Moderate
Pindolol	>90	≈90	No	Moderate
Propranolol	>90	30‐70	Yes	High
Sotalol	>80^b^	≈90	No	Low
Timolol	>90	≈75	Yes	Low‐moderate

a From.^34^

b From.^21^

For CV < 40% (low or moderate variability) the median AUC ratio for single‐dose studies was 2.4 (range 1.1‐4.6; n = 28); for CV > 40% (high variability) the median AUC ratio for single‐dose studies was 5.8 (range 2.0‐46; n = 29).

For CV < 40% (low or moderate variability) the median AUC ratio for steady‐state studies was 2.0 (range 1.7‐4.4; n = 6); for CV > 40% (high variability) the median AUC ratio for steady‐state studies was 5.1 (range 1.9‐29.5; n = 11). The highest ratio was found in metoprolol.

## 4 DISCUSSION

Our main finding is a considerable interindividual pharmacokinetic variability in a subset of β‐blockers used in the treatment of several cardiovascular conditions. In particular, carvedilol, metoprolol, nebivolol, and propranolol showed a high frequency of studies with high pharmacokinetic variability (CV > 40%), whereas atenolol, bisoprolol, labetalol, nadolol, pindolol, and sotalol showed low or moderate variability (CV < 40%).

The optimal β‐blocker provides the desired lowered risk of myocardial infarction and sudden cardiac death as well as minimizes the number of adverse events, both minor and major.^13^ In clinical use β‐1‐selective and nonselective β‐blockers are similar with regards to antihypertensive, antiarrhythmic, and antianginal effect, when adequately dosed.^14^ When deciding on which β‐blocker to prescribe a patient in the clinic, known contradictions should be asserted eg hepatic impairment, renal insufficiency, diabetes, and obstructive lung disease. In addition, patients who are prescribed β‐blockers often have comorbidities and are therefore treated with multiple drugs that may have a pharmacokinetic interaction with a subsequent change in drug concentration.^15^ This interaction has been shown with metoprolol and the SSRI fluoxetine (CYP2D6 inhibitor), which increase the concentration of metoprolol thus inducing bradycardia.^16^

As shown in this review, the AUC varies between individuals receiving the same dosage of eg metoprolol with the most extreme ratio of 30‐fold from highest to lowest AUC.^17^

Differences in pharmacokinetic parameters of β‐blockers are apparent in patients when comparing healthy individuals with patients with kidney or liver disease, but differences are also present among apparently comparable healthy subjects as seen in this review and can largely be explained by the four ADME phases:

Bioavailability of β‐blockers varies in part because of first–pass hepatic elimination resulting in a varying oral bioavailability, see Table 3. This is further complicated by food–induced changes in the bioavailability, where food induces a 20% reduction in the bioavailability of atenolol, while it tends to enhance the bioavailability in metoprolol (40%) and propranolol (53%).^18^, ^19^, ^20^ In the pharmacokinetic studies in this review the participants were generally fasting prior to receiving the test formulation in single‐dose studies, while the participants in steady‐state studies also abstained from food‐intake prior to receiving the test formulation.

The metabolism and elimination of β‐blockers depends partly on whether the β‐blocker is lipophilic, in which case it is almost completely metabolized by the liver, or hydrophilic, in which case elimination is mainly dependent on glomerular filtration.^21^ In this review, hydrophilic β‐blockers (atenolol, nadolol, sotalol) showed a generally lower number of studies where CV% for AUC was > 40%. As atenolol, nadolol, and sotalol avoid first–pass metabolism in the liver, the interindividual pharmacokinetic variation in bioavailability may not be as extensive in hydrophilic β‐blockers.^22^ On the contrary, all the β‐blockers showing high pharmacokinetic variability in our review are metabolized primarily in the liver mediated by the CYP2D6 enzyme.

The liver metabolism has been shown to be highly dependent on the phenotype of the individual, where poor metabolizers, in theory, are more prone to experience adverse events compared to extensive metabolizers, because of a lower needed dosage.^8^ but, in practice, this theory has not been validated in studies on metoprolol.^23^, ^24^ This discrepancy could be attributed to differences in the sympathetic tone in patients or a rather flat dose–response curve in some β‐blockers.^22^ We have compared extended release formulations with regular tablets of metoprolol in steady‐state studies and found the median CV for AUC to be lower for extended release formulations (54% vs. 72%), but no substantial differences were seen for the median CV for C_max_. But as this is only descriptive, a meta‐analysis of the studies should be done to make any statistical conclusions on the effect of extended release formulations’ effect on the pharmacokinetics of β‐blockers.

The ratios for AUC in both single‐dose and steady‐state studies corresponded with the variability seen for CV% for AUC. The interpatient variability was expressed as the CV for AUC, which likely is the most relevant parameter for clinical use of the β‐blockers. Rowland and Tozer showed the AUC to infinity for a single oral dose to be equal to the AUC to time in steady‐state studies, and thus we included results from single‐dose studies on AUC in Figure 1: ${\text{AUC}}_{\text{SS},0-\tau }={\text{AUC}}_{\text{SD},0-\infty }$.^12^

The pharmacokinetic results in this review, obtained from several studies, are primarily deducted from young to middle aged healthy male individuals, thereby potentially underestimating the true potentially greater pharmacokinetic differences found in elderly patients, who are the main recipients of β‐blockers and often are in medical treatment for other comorbidities. Only a few studies included in this review subdivided the volunteers in a “young” and “elderly” category, but pharmacokinetic results from these studies point in different directions. Castleden et al showed an effect of aging on the pharmacokinetics of propranolol both in single and steady‐state doses: the elderly not only had a 2.3 times higher C_max_ in the single‐dose study, but the steady‐state study showed an overall plasma concentration of 3.1 times higher compared to the young.^25^ Castleden et al explains this difference as a result of reduced hepatic blood flow and first–pass extraction in the elderly, which is important as a majority of prescriptions are for the elderly population.

More than 100 different CYP2D6 gene polymorphisms have been identified since the 1970s.^8^ These genetic variants form the basis of the four phenotypes: ultrarapid, extensive, intermediate, and poor metabolizers of medication metabolized by the CYP2D6 enzyme. The pharmacokinetic differences in patients cause different bioavailability between patients even though the same drug dosage has been administered; consequently, extensive metabolizers of the CYP2D6 enzyme will need markedly higher doses of metoprolol than poor metabolizers to obtain the same plasma concentration. Blake et al compared the C_max_ and AUC of metoprolol in ultrarapid metabolizers and poor metabolizers in a pooled analysis and found a 5.3‐fold (C_max_/dose; 90% confidence interval (CI): 3.6‐6.9‐fold; *P *< 0.0001) and 13‐fold (AUC/dose; 90% CI: 9.4‐19.7‐fold) difference, respectively.^11^

The clinical implications of our results are the substantial differences in interpatient pharmacokinetic variability in different β‐blockers. Administration of β‐blockers with high pharmacokinetic variability should be more cautiously initiated, as the daily maintenance dose varies more from patient to patient. Combined with the membrane–stabilizing effect of nadolol, the low pharmacokinetic variation may explain why nadolol is the most efficient β‐blocker to reduce life–threatening arrhythmic events in LQTS patients.^26^ As illustrated in Figure 1, the pharmacokinetic variability of carvedilol is found to be approximately 60%, which is in conjunction with findings in Packer et al's study on the lowering morbidity and mortality of carvedilol in patients with heart failure, where a steady state dosage of 45 ± 27 mg (60% CV) of carvedilol was seen.^27^

Difference in β‐1 selectivity/nonselectivity among β‐blockers and the potency of said β‐blocker are important factors when deciding on β‐blocker to a patient, comorbidities considered.^28^ Genetic polymorphisms for the β‐1 adrenoceptor have been identified and investigated in relation to pharmacodynamic variability, but the no clear relation has been shown.^29^ Receptor antagonism of the adrenergic β‐1 receptor induces lowered sympathetic activation thereby lowering the heart rate and contractility of the heart, while antagonism of the β‐2 receptor may induce vasoconstriction of smooth muscle cells in the bronchi and blood vessels, why asthma patients should avoid nonselective β‐blockers.^30^ Nonselective β‐blockers may also mask symptoms of hypoglycemia in diabetics.^30^

A major limitation of our findings is the number of studies conducted on the different types of β‐blockers; the β‐blockers that have been on the market for the longest time have had a large amount of studies conducted compared to newer as well as rarely used β‐blockers. Metoprolol and propranolol are often used in comparative studies with newer formulations.

Factors such as sex, obesity, and ethnicity have also been proved to be of importance in the pharmacokinetics.^31^, ^32^ Ueno et al have previously reviewed sex–specific differences in pharmacokinetic studies of metoprolol and propranolol, where females had greater drug exposure than males in part due to different body composition.^31^ The studies included in our review were mainly conducted on healthy Caucasian male volunteers with a BMI of 20‐25, and the investigation of sex–specific differences in the pharmacokinetics was not possible in the current study. The subjects were typically matched on sex, body weight, age, and health status, and crossover studies were generally conducted. A few studies differentiated on CYP2D6‐status, while in most studies it was not investigated. As the findings from this review are related to healthy volunteers, the known influence of eg age, sex, genotype, and comorbidities in the elderly will in theory further increase the interindividual pharmacokinetic variability.

Future research should focus on the matter that, as genetic testing is becoming more common, available data obtained from here should somehow be included in the clinicians’ considerations when prescribing a patient a β‐blocker, eg if the patient is a poor metabolizer of metoprolol thereby reducing the initial dosage in combination with the patient's general characteristics (sex, age, BMI).^33^

The beneficial effects of β‐blockers in eligible patients are strong and the impact has caused β‐blockers to be considered one of the greatest pharmacotherapeutic advances of the 20th century. The pharmacology is widely understood, but the individual response is often unpredictable and should lead to a careful up‐titration of the medication.

In conclusion, we showed how the pharmacokinetic variability between patients vary markedly in different β‐blockers, and we showed how the ratios between highest and lowest steady‐state concentrations differ with the highest ratio in metoprolol being 30.

By combining known pharmacokinetic information with pharmacogenetic findings (CYP‐status for lipophilic β‐blockers) with pharmacodynamics (pulse or blood pressure) and the individual patient's characteristics, a more personalized treatment may be obtained in the future, minimizing adverse events or lack of effect.

## DISCLOSURE

There are no competing interests to declare.
