# Supplementary material for: Pharmacokinetic variability of beta‐adrenergic blocking agents used in cardiology
Source: Pharmacol Res Perspect. 2019 Jul 12;7(4):e00496. doi: 10.1002/prp2.496 (PMC6624454; doi:10.1002/prp2.496)
Supplement: Supplementary file 1 [file PRP2-7-e00496-s001.docx]

**SUPPORTING INFORMATION**

**References for Table 1 and Table 2:**

35. Brown HC, Carruthers SG, Johnston GD, et al. Clinical pharmacologic observations on atenolol, a beta-adrenoceptor blocker. *Clin Pharmacol Ther*. 1976;20(5):524-534.

36. Mason WD, Winer N, Kochak G, Cohen I, Bell R. Kinetics and absolute bioavailability of atenolol. *Clin Pharmacol Ther*. 1979;25(4):408-415.

37. Schäfer-Korting M, Kirch W, Axthelm T, Köhler H, Mutschler E. Atenolol interaction with aspirin, allopurinol, and ampicillin. *Clin Pharmacol Ther*. 1983;33(3):283-288.

38. Tateishi T, Nakashima H, Shitou T, et al. Effect of diltiazem on the pharmacokinetics of propranolol, metoprolol and atenolol. *Eur J Clin Pharmacol*. 1989;36(1):67-70.

39. Boyd RA, Chin SK, Don-Pedro O, Williams RL, Giacomini KM. The pharmacokinetics of the enantiomers of atenolol. *Clin Pharmacol Ther*. 1989;45(4):403-410.

40. Mehvar R, Gross ME, Kreamer RN. Pharmacokinetics of atenolol enantiomers in humans and rats. *J Pharm Sci*. 1990;79(10):881-885.

41. Ferry N, Bernard N, Pozet N, et al. The effect of infinitesimal drug dilutions on the pharmacokinetics of nalidixic acid and atenolol. *Br J Clin Pharmacol*. 1991;32(1):39-44.

42. Greenblatt DJ, Scavone JM, Harmatz JS, Engelhardt N, Shader RI. Cognitive effects of beta-adrenergic antagonists after single doses: pharmacokinetics and pharmacodynamics of propranolol, atenolol, lorazepam, and placebo. *Clin Pharmacol Ther*. 1993;53(5):577-584.

43. Irshaid YM, Rawashdeh NM, Awwadi FF, Kato MK. Comparative pharmacokinetics of two brands of atenolol following a single oral administration. *Int J Clin Pharmacol Ther*. 1996;34(10):457-461.

44. Czendlik CH, Sioufi A, Preiswerk G, Howald H. Pharmacokinetic and pharmacodynamic interaction of single doses of valsartan and atenolol. *Eur J Clin Pharmacol*. 1997;52(6):451-459.

45. Martins ML, Pierossi MA, Moraes LA, et al. Comparative bioavailability of two atenolol tablet formulations in healthy male volunteers after a single dose administration. *Int J Clin Pharmacol Ther*. 1997;35(8):324-328.

46. Rojanasthien N, Manorot M, Kumsorn B. Bioequivalence study of generic atenolol tablets in healthy Thai volunteers. *J Med Assoc Thail Chotmaihet Thangphaet*. 1999;82(9):907-914.

47. Wang XM, Yu XY, Lin SG. Pharmacokinetics of atenolol enantiomers in 12 Chinese healthy men. *Zhongguo Yao Li Xue Bao*. 1999;20(4):367-370.

48. Niopas I, Daftsios AC, Xanthakis I, Nikolaidis N, Njau SN. Bioequivalence of two tablet formulations of atenolol after single oral administration in healthy volunteers. *Arzneimittelforschung*. 2000;50(3):243-247. doi:10.1055/s-0031-1300193

49. Wójcicki J, Wojciechowski G, Wójcicki M, et al. Pharmacokinetics of propranolol and atenolol in patients after partial gastric resection: a comparative study. *Eur J Clin Pharmacol*. 2000;56(1):75-79.

50. Drozdzik M, Domanski L, Wojcicki J, Pudlo A, Machoy P. Effect of unilateral nephrectomy on the pharmacokinetics of atenolol in humans. *J Clin Pharmacol*. 2003;43(5):524-528.

51. Telatyńska B, Wójcicki J, Droździk M, Gawrońska-Szklarz B, Sulzyc-Bielicka V, Sterna R. Comparative pharmacokinetics of propranolol and atenolol in primary hyperlipidemia. *Pol J Pharmacol*. 2003;55(1):81-89.

52. Najib NM, Idkaidek N, Adel A, et al. Comparative bioavailability of two brands of atenolol 100 mg tablets (Tensotin and Tenormin) in healthy human volunteers. *Biopharm Drug Dispos*. 2005;26(1):1-5. doi:10.1002/bdd.416

53. Lilja JJ, Juntti-Patinen L, Neuvonen PJ. Effect of rifampicin on the pharmacokinetics of atenolol. *Basic Clin Pharmacol Toxicol*. 2006;98(6):555-558. doi:10.1111/j.1742-7843.2006.pto_379.x

54. Patel A, Shah T, Shah G, et al. Preservation of bioavailability of ingredients and lack of drug-drug interactions in a novel five-ingredient polypill (polycap): a five-arm phase I crossover trial in healthy volunteers. *Am J Cardiovasc Drugs Drugs Devices Interv*. 2010;10(2):95-103. doi:10.2165/11532170-000000000-00000

55. Chang MJ, Shin WG. Comparative pharmacokinetics and bioequivalence of two 50 mg atenolol tablet formulations in healthy Korean male volunteers. *Arzneimittelforschung*. 2012;62(9):410-413. doi:10.1055/s-0032-1314853

56. Azizi M, Blanchard A, Charbit B, et al. Effect of contrasted sodium diets on the pharmacokinetics and pharmacodynamic effects of renin-angiotensin system blockers. *Hypertension*. 2013;61(6):1239-1245. doi:10.1161/HYPERTENSIONAHA.113.01196

57. Ishizaki T, Oyama Y, Suganuma T, et al. A dose ranging study of atenolol in hypertension: fall in blood pressure and plasma renin activity, beta-blockade and steady-state pharmacokinetics. *Br J Clin Pharmacol*. 1983;16(1):17-25.

58. Darmansjah I, Wong E, Setiawati A, et al. Pharmacokinetic and pharmacodynamic properties of controlled release (CR/ZOK) metoprolol in healthy Oriental subjects: a comparison with conventional formulations of metoprolol and atenolol. *J Clin Pharmacol*. 1990;30(2 Suppl):S39-45.

59. Elliott HL, Meredith PA, McNally C, Reid JL. The interactions between nisoldipine and two beta-adrenoceptor antagonists--atenolol and propranolol. *Br J Clin Pharmacol*. 1991;32(3):379-385.

60. Andrawis NS, Battle MM, Klamerus KJ, et al. A pharmacokinetic and pharmacodynamic study of the potential drug interaction between tasosartan and atenolol in patients with stage 1 and 2 essential hypertension. *J Clin Pharmacol*. 2000;40(3):231-241.

61. Bianchetti G, Thiercelin JF, Thenot JP. Pharmacokinetics of betaxolol in middle aged patients. *Eur J Clin Pharmacol*. 1986;31(2):231-233.

62. Rey E, Jammet P, d’Athis P, et al. Effect of cimetidine on the pharmacokinetics of the new beta-blocker betaxolol. *Arzneimittelforschung*. 1987;37(8):953-956.

63. Wellstein A, Palm D, Belz GG, Leopold G, Bühring KU, Pabst J. Concentration kinetics of propranolol, bisoprolol, and atenolol in humans assessed with chemical detection and a subtype-selective beta-adrenoceptor assay. *J Cardiovasc Pharmacol*. 1986;8 Suppl 11:S41-45.

64. Dutta A, Lanc R, Begg E, et al. Dose proportionality of bisoprolol enantiomers in humans after oral administration of the racemate. *J Clin Pharmacol*. 1994;34(8):829-836.

65. Deroubaix X, Lins RL, Lens S, et al. Comparative bioavailability of a metoprolol controlled release formulation and a bisoprolol normal release tablet after single oral dose administration in healthy volunteers. *Int J Clin Pharmacol Ther*. 1996;34(2):61-70.

66. Bus-Kwasnik K, Ksycinska H, Les A, et al. Bioequivalence and pharmacokinetics of two 10-mg bisoprolol formulations as film-coated tablets in healthy white volunteers: a randomized, crossover, open-label, 2-period, single-dose, fasting study. *Int J Clin Pharmacol Ther*. 2012;50(12):909-919. doi:10.5414/CP201726

67. Tjandrawinata RR, Setiawati E, Yunaidi DA, Santoso ID, Setiawati A, Susanto LW. Bioequivalence study of two formulations of bisoprolol fumarate film-coated tablets in healthy subjects. *Drug Des Devel Ther*. 2012;6:311-316. doi:10.2147/DDDT.S36567

68. Tjandrawinata RR, Setiawati E, Yunaidi DA, Santoso ID, Setiawati A, Susanto LW. Bioequivalence study of 2 formulations of film-coated tablets containing a fixed dose combination of bisoprolol fumarate 5 mg and hydrochlorothiazide 6.25 mg in healthy subjects. *Drug Res*. 2013;63(5):243-249. doi:10.1055/s-0033-1334922

69. Buś-Kwaśnik K, Rudzki PJ, Ksycińska H, et al. Bioequivalence study of 2.5 mg film-coated bisoprolol tablets in healthy volunteers. *Kardiol Pol*. 2017;75(1):48-54. doi:10.5603/KP.a2016.0106

70. van Gelderen M, Stölzel M, Meijer J, Kerbusch V, Collins C, Korstanje C. An Exploratory Study in Healthy Male Subjects of the Mechanism of Mirabegron-Induced Cardiovascular Effects. *J Clin Pharmacol*. 2017;57(12):1534-1544. doi:10.1002/jcph.952

71. Kirch W, Rose I, Klingmann I, Pabst J, Ohnhaus EE. Interaction of bisoprolol with cimetidine and rifampicin. *Eur J Clin Pharmacol*. 1986;31(1):59-62.

72. Kirch W, Rose I, Demers HG, Leopold G, Pabst J, Ohnhaus EE. Pharmacokinetics of bisoprolol during repeated oral administration to healthy volunteers and patients with kidney or liver disease. *Clin Pharmacokinet*. 1987;13(2):110-117.

73. Gehr TW, Tenero DM, Boyle DA, Qian Y, Sica DA, Shusterman NH. The pharmacokinetics of carvedilol and its metabolites after single and multiple dose oral administration in patients with hypertension and renal insufficiency. *Eur J Clin Pharmacol*. 1999;55(4):269-277.

74. do Carmo Borges NC, Mendes GD, de Oliveira Silva D, Marcondes Rezende V, Barrientos-Astigarraga RE, De Nucci G. Quantification of carvedilol in human plasma by high-performance liquid chromatography coupled to electrospray tandem mass spectrometry: application to bioequivalence study. *J Chromatogr B Analyt Technol Biomed Life Sci*. 2005;822(1-2):253-262. doi:10.1016/j.jchromb.2005.06.012

75. Portolés A, Filipe A, Almeida S, Terleira A, Vallée F, Vargas E. Bioequivalence Study of Two Different Tablet Formulations of Carvedilol in Healthy Volunteers. *Arzneimittelforschung*. 2011;55(04):212-217. doi:10.1055/s-0031-1296847

76. Henderson LS, Tenero DM, Campanile AM, Baidoo CA, Danoff TM. Ethanol does not alter the pharmacokinetic profile of the controlled-release formulation of carvedilol. *J Clin Pharmacol*. 2007;47(11):1358-1365. doi:10.1177/0091270007307244

77. Liu X, Wang B, Yuan G, Guo R. Comparison of different pharmacodynamic models for pharmacokinetic-pharmacodynamic (PK-PD) modeling of carvedilol. *Yao Xue Xue Bao*. 2009;44(4):406-411.

78. Stout SM, Nielsen J, Bleske BE, et al. The impact of paroxetine coadministration on stereospecific carvedilol pharmacokinetics. *J Cardiovasc Pharmacol Ther*. 2010;15(4):373-379. doi:10.1177/1074248410372926

79. Agrawal SS, Aggarwal A. Randomised, cross-over, comparative bioavailability trial of matrix type transdermal drug delivery system (TDDS) of carvedilol and hydrochlorothiazide combination in healthy human volunteers: A pilot study. *Contemp Clin Trials*. 2010;31(4):272-278. doi:10.1016/j.cct.2010.03.013

80. Liu Y, Lu C, Chen Q, et al. Bioequivalence and pharmacokinetic evaluation of two tablet formulations of carvedilol 25-mg: a single-dose, randomized-sequence, open-label, two-way crossover study in healthy Chinese male volunteers. *Drug Res*. 2013;63(2):74-78. doi:10.1055/s-0032-1331768

81. Kim YH, Choi HY, Noh Y-H, et al. Dose proportionality and pharmacokinetics of carvedilol sustained-release formulation: a single dose-ascending 10-sequence incomplete block study. *Drug Des Devel Ther*. 2015;9:2911-2918. doi:10.2147/DDDT.S86168

82. Kanto J, Allonen H, Kleimola T, Mäntylä R. Pharmacokinetics of labetalol in healthy volunteers. *Int J Clin Pharmacol*. 1981;19(1):41-44.

83. Nyberg G, Hansson R, Tietz F. Single-dose pharmacokinetics of labetalol in healthy young men. *Acta Med Scand Suppl*. 1982;665:67-73.

84. McNeil JJ, Anderson AE, Louis WJ, Raymond K. Labetalol steady-state pharmacokinetics in hypertensive patients. *Br J Clin Pharmacol*. 1982;13(1 Suppl):75S-80S.

85. Fujimura A, Ohashi K, Tsuru M, Ebihara A, Kondo K. Clinical pharmacology of dilevalol (I). Comparison of the pharmacokinetic and pharmacodynamic properties of dilevalol and labetalol after a single oral administration in healthy subjects. *J Clin Pharmacol*. 1989;29(7):635-642.

86. Rasmussen BB, Larsen LS, Senderovitz T. Pharmacokinetic interaction studies of atosiban with labetalol or betamethasone in healthy female volunteers. *BJOG Int J Obstet Gynaecol*. 2005;112(11):1492-1499. doi:10.1111/j.1471-0528.2005.00735.x

87. Johnson JA, Akers WS, Herring VL, Wolfe MS, Sullivan JM. Gender differences in labetalol kinetics: importance of determining stereoisomer kinetics for racemic drugs. *Pharmacotherapy*. 2000;20(6):622-628.

88. Awni WM, Skaar DJ, Schwenk MH, Sirgo MA, Plachetka JR, Matzke GR. Interindividual and intraindividual variability in labetalol pharmacokinetics. *J Clin Pharmacol*. 1988;28(4):344-349.

89. Tenero DM, Bottorff MB, Given BD, et al. Pharmacokinetics and pharmacodynamics of dilevalol. *Clin Pharmacol Ther*. 1989;46(6):648-656.

90. Myers MG, Thiessen JJ. Metoprolol kinetics and dose response in hypertensive patients. *Clin Pharmacol Ther*. 1980;27(6):756-762.

91. Quarterman CP, Kendall MJ, Jack DB. The effect of age on the pharmacokinetics of metoprolol and its metabolites. *Br J Clin Pharmacol*. 1981;11(3):287-294.

92. Regårdh CG, Lundborg P, Persson BA. The effect of antacid, metoclopramide, and propantheline on the bioavailability of metoprolol and atenolol. *Biopharm Drug Dispos*. 1981;2(1):79-87.

93. Jack DB, Quarterman CP, Zaman R, Kendall MJ. Variability of beta-blocker pharmacokinetics in young volunteers. *Eur J Clin Pharmacol*. 1982;23(1):37-42.

94. Briant RH, Dorrington RE, Ferry DG, Paxton JW. Bioavailability of metoprolol in young adults and the elderly, with additional studies on the effects of metoclopramide and probanthine. *Eur J Clin Pharmacol*. 1983;25(3):353-356.

95. Kendall MJ, Jack DB, Quarterman CP, Smith SR, Zaman R. Beta-adrenoceptor blocker pharmacokinetics and the oral contraceptive pill. *Br J Clin Pharmacol*. 1984;17 Suppl 1:87S-89S.

96. Warrington SJ, Barclay SP, John VA, Shotton PA, Good W. Comparison of single-dose pharmacokinetic and pharmacodynamic properties of two metoprolol Oros systems with different initial zero-order release rates. *Br J Clin Pharmacol*. 1985;19 Suppl 2:225S-230S.

97. Lloyd P, John VA, Signy M, Smith SE. The effect of impaired renal function on the pharmacokinetics of metoprolol after single administration of a 14/190 metoprolol OROS system. *Am Heart J*. 1990;120(2):478-482.

98. de Stoppelaar FM, Stolk LM, Beysens AJ, Stappers JL, Gorgels AP. The relative bioavailability of metoprolol following oral and rectal administration to volunteers and patients. *Pharm World Sci PWS*. 1999;21(5):233-238.

99. Bauer LA, Horn JR, Maxon MS, Easterling TR, Shen DD, Strandness DE. Effect of metoprolol and verapamil administered separately and concurrently after single doses on liver blood flow and drug disposition. *J Clin Pharmacol*. 2000;40(5):533-543.

100. Hemeryck A, Lefebvre RA, De Vriendt C, Belpaire FM. Paroxetine affects metoprolol pharmacokinetics and pharmacodynamics in healthy volunteers. *Clin Pharmacol Ther*. 2000;67(3):283-291. doi:10.1067/mcp.2000.104788

101. Donovan JM, Stypinski D, Stiles MR, Olson TA, Burke SK. Drug interactions with colesevelam hydrochloride, a novel, potent lipid-lowering agent. *Cardiovasc Drugs Ther Spons Int Soc Cardiovasc Pharmacother*. 2000;14(6):681-690.

102. Hamelin BA, Bouayad A, Méthot J, et al. Significant interaction between the nonprescription antihistamine diphenhydramine and the CYP2D6 substrate metoprolol in healthy men with high or low CYP2D6 activity. *Clin Pharmacol Ther*. 2000;67(5):466-477. doi:10.1067/mcp.2000.106464

103. Burke SK, Amin NS, Incerti C, Plone MA, Lee JW. Sevelamer hydrochloride (Renagel), a phosphate-binding polymer, does not alter the pharmacokinetics of two commonly used antihypertensives in healthy volunteers. *J Clin Pharmacol*. 2001;41(2):199-205.

104. Werner U, Werner D, Rau T, Fromm MF, Hinz B, Brune K. Celecoxib inhibits metabolism of cytochrome P450 2D6 substrate metoprolol in humans. *Clin Pharmacol Ther*. 2003;74(2):130-137. doi:10.1016/S0009-9236(03)00120-6

105. Sharma A, Pibarot P, Pilote S, et al. Modulation of metoprolol pharmacokinetics and hemodynamics by diphenhydramine coadministration during exercise testing in healthy premenopausal women. *J Pharmacol Exp Ther*. 2005;313(3):1172-1181. doi:10.1124/jpet.104.081109

106. Ravishankar H, Patil P, Samel A, Petereit H-U, Lizio R, Iyer-Chavan J. Modulated release metoprolol succinate formulation based on ionic interactions: in vivo proof of concept. *J Control Release Off J Control Release Soc*. 2006;111(1-2):65-72. doi:10.1016/j.jconrel.2005.12.007

107. Chen M-L, Straughn AB, Sadrieh N, et al. A modern view of excipient effects on bioequivalence: case study of sorbitol. *Pharm Res*. 2007;24(1):73-80. doi:10.1007/s11095-006-9120-4

108. Aqil M, Ali A, Sultana Y, Saha N. Comparative bioavailability of metoprolol tartrate after oral and transdermal administration in healthy male volunteers. *Clin Drug Investig*. 2007;27(12):833-839.

109. Jin SK, Chung HJ, Chung MW, et al. Influence of CYP2D6*10 on the pharmacokinetics of metoprolol in healthy Korean volunteers. *J Clin Pharm Ther*. 2008;33(5):567-573. doi:10.1111/j.1365-2710.2008.00945.x

110. Seeringer A, Brockmöller J, Bauer S, Kirchheiner J. Enantiospecific pharmacokinetics of metoprolol in CYP2D6 ultra-rapid metabolizers and correlation with exercise-induced heart rate. *Eur J Clin Pharmacol*. 2008;64(9):883-888. doi:10.1007/s00228-008-0504-8

111. Wang Y, Zhou L, Dutreix C, et al. Effects of imatinib (Glivec) on the pharmacokinetics of metoprolol, a CYP2D6 substrate, in Chinese patients with chronic myelogenous leukaemia. *Br J Clin Pharmacol*. 2008;65(6):885-892. doi:10.1111/j.1365-2125.2008.03150.x

112. Turpault S, Brian W, Van Horn R, et al. Pharmacokinetic assessment of a five-probe cocktail for CYPs 1A2, 2C9, 2C19, 2D6 and 3A. *Br J Clin Pharmacol*. 2009;68(6):928-935. doi:10.1111/j.1365-2125.2009.03548.x

113. Sharma A, Pibarot P, Pilote S, et al. Toward optimal treatment in women: the effect of sex on metoprolol-diphenhydramine interaction. *J Clin Pharmacol*. 2010;50(2):214-225. doi:10.1177/0091270009340417

114. Parker RB, Soberman JE. Effects of paroxetine on the pharmacokinetics and pharmacodynamics of immediate-release and extended-release metoprolol. *Pharmacotherapy*. 2011;31(7):630-641. doi:10.1592/phco.31.7.630

115. Inoue S, Shimizu M, Arita K, Akimoto K. The effect of AST-120 on the single-dose pharmacokinetics of metoprolol extended-release tablets in healthy subjects. *Drug Metabol Drug Interact*. 2014;29(2):115-121. doi:10.1515/dmdi-2013-0063

116. Krauwinkel W, Dickinson J, Schaddelee M, et al. The effect of mirabegron, a potent and selective β3-adrenoceptor agonist, on the pharmacokinetics of CYP2D6 substrates desipramine and metoprolol. *Eur J Drug Metab Pharmacokinet*. 2014;39(1):43-52. doi:10.1007/s13318-013-0133-1

117. Cho D-Y, Bae SH, Lee JK, et al. Effect of the potent CYP2D6 inhibitor sarpogrelate on the pharmacokinetics and pharmacodynamics of metoprolol in healthy male Korean volunteers. *Xenobiotica Fate Foreign Compd Biol Syst*. 2015;45(3):256-263. doi:10.3109/00498254.2014.967824

118. Garimella T, Tao X, Sims K, et al. Effects of a Fixed-Dose Co-Formulation of Daclatasvir, Asunaprevir, and Beclabuvir on the Pharmacokinetics of a Cocktail of Cytochrome P450 and Drug Transporter Substrates in Healthy Subjects. *Drugs RD*. 2018;18(1):55-65. doi:10.1007/s40268-017-0222-8

119. Larsson M, Landahl S, Lundborg P, Regårdh CG. Pharmacokinetics of metoprolol in healthy, elderly, non-smoking individuals after a single dose and two weeks of treatment. *Eur J Clin Pharmacol*. 1984;27(2):217-222.

120. Kelly JG, Salem SA, Kinney CD, Shanks RG, McDevitt DG. Effects of ranitidine on the disposition of metoprolol. *Br J Clin Pharmacol*. 1985;19(2):219-224.

121. Wagner F, Jähnchen E, Trenk D, et al. Severe complications of antianginal drug therapy in a patient identified as a poor metabolizer of metoprolol, propafenone, diltiazem, and sparteine. *Klin Wochenschr*. 1987;65(24):1164-1168.

122. Jonkers R, van Boxtel CJ, Koopmans RP, Oosterhuis B. A nonsteady-state agonist antagonist interaction model using plasma potassium concentrations to quantify the beta-2 selectivity of beta blockers. *J Pharmacol Exp Ther*. 1989;249(1):297-302.

123. Lesko LJ, Offman E, Brew CT, et al. Evaluation of the Potential for Drug Interactions With Patiromer in Healthy Volunteers. *J Cardiovasc Pharmacol Ther*. 2017;22(5):434-446. doi:10.1177/1074248417691135

124. Regårdh CG, Johnsson G, Jordö L, Sölvell L. Comparative bioavailability and effect studies on metoprolol administered as ordinary and slow-release tablets in single and multiple doses. *Acta Pharmacol Toxicol (Copenh)*. 1975;36(Suppl 5):45-58.

125. Jordö L, Attman PO, Aurell M, Johansson L, Johnsson G, Regårdh CG. Pharmacokinetic and pharmacodynamic properties of metoprolol in patients with impaired renal function. *Clin Pharmacokinet*. 1980;5(2):169-180.

126. Kendall MJ, Jack DB, Woods KL, Laugher SJ, Quarterman CP, John VA. Comparison of the pharmacodynamic and pharmacokinetic profiles of single and multiple doses of a commercial slow-release metoprolol formulation with a new Oros delivery system. *Br J Clin Pharmacol*. 1982;13(3):393-398.

127. Gangji D, Juvent M, Niset G, et al. Study of the influence of nifedipine on the pharmacokinetics and pharmacodynamics of propranolol, metoprolol and atenolol. *Br J Clin Pharmacol*. 1984;17 Suppl 1:29S-35S.

128. Rigby JW, Scott AK, Hawksworth GM, Petrie JC. A comparison of the pharmacokinetics of atenolol, metoprolol, oxprenolol and propranolol in elderly hypertensive and young healthy subjects. *Br J Clin Pharmacol*. 1985;20(4):327-331.

129. Blomqvist I, Westergren G, Sandberg A, Jonsson UE, Lundborg P. Pharmacokinetics and pharmacodynamics of controlled-release metoprolol: a comparison with atenolol. *Eur J Clin Pharmacol*. 1988;33 Suppl:S19-24.

130. Oosterhuis B, Jonkman JH, Kerkhof FA. Pharmacokinetic and pharmacodynamic comparison of a new controlled-release formulation of metoprolol with a traditional slow-release formulation. *Eur J Clin Pharmacol*. 1988;33 Suppl:S15-18.

131. Sandberg A, Blomqvist I, Jonsson UE, Lundborg P. Pharmacokinetic and pharmacodynamic properties of a new controlled-release formulation of metoprolol: a comparison with conventional tablets. *Eur J Clin Pharmacol*. 1988;33 Suppl:S9-14.

132. Toon S, Davidson EM, Garstang FM, Batra H, Bowes RJ, Rowland M. The racemic metoprolol H2-antagonist interaction. *Clin Pharmacol Ther*. 1988;43(3):283-289.

133. Kirch W, Santos SR, Geller M, Mönig H, Stenzel J, Ohnhaus EE. Influence of nitrendipine and verapamil on plasma levels, urinary excretion, and beta-blocking effect of metoprolol. *Cardiovasc Drugs Ther Spons Int Soc Cardiovasc Pharmacother*. 1988;2(2):205-209.

134. Lee YT, Liau CS, Wong EC, Chen WJ, Chen MF, Chen CC. Pharmacokinetic and pharmacodynamic comparison of conventional and controlled release formulations of metoprolol [correction of motoprolol] in healthy Chinese subjects. *Cardiovasc Drugs Ther Spons Int Soc Cardiovasc Pharmacother*. 1989;3(4):529-533.

135. Feliciano NR, Bouvet AA, Redalieu E, et al. Pharmacokinetic and pharmacodynamic comparison of an osmotic release oral metoprolol tablet and the metoprolol conventional tablet. *Am Heart J*. 1990;120(2):483-489.

136. Dimenäs E, Kerr D, Macdonald I. Beta-adrenoceptor blockade and CNS-related subjective symptoms: a randomized, double-blind, placebo-controlled comparison of metoprolol CR/ZOK, atenolol and propranolol LA in healthy subjects. *J Clin Pharmacol*. 1990;30(2 Suppl):S103-107.

137. Lücker P, Moore G, Wieselgren I, Olofsson B, Bergstrand R. Pharmacokinetic and pharmacodynamic comparison of metoprolol CR/ZOK once daily with conventional tablets once daily and in divided doses. *J Clin Pharmacol*. 1990;30(2 Suppl):S17-27.

138. Sandberg A, Abrahamsson B, Svenheden A, Olofsson B, Bergstrand R. Steady-state bioavailability and day-to-day variability of a multiple-unit (CR/ZOK) and a single-unit (OROS) delivery system of metoprolol after once-daily dosing. *Pharm Res*. 1993;10(1):28-34.

139. Aberg J, Abrahamsson B, Grind M, Nyberg G, Olofsson B. Bioequivalence, pharmacokinetic and pharmacodynamic response to combined extended release formulations of felodipine and metoprolol in healthy volunteers. *Eur J Clin Pharmacol*. 1997;52(6):471-477.

140. Luzier AB, Killian A, Wilton JH, Wilson MF, Forrest A, Kazierad DJ. Gender-related effects on metoprolol pharmacokinetics and pharmacodynamics in healthy volunteers. *Clin Pharmacol Ther*. 1999;66(6):594-601. doi:10.1053/cp.1999.v66.103400001

141. Damy T, Pousset F, Caplain H, Hulot J-S, Lechat P. Pharmacokinetic and pharmacodynamic interactions between metoprolol and dronedarone in extensive and poor CYP2D6 metabolizers healthy subjects. *Fundam Clin Pharmacol*. 2004;18(1):113-123.

142. Schäfer-Korting M, Bach N, Knauf H, Mutschler E. Pharmacokinetics of nadolol in healthy subjects. *Eur J Clin Pharmacol*. 1984;26(1):125-127.

143. Srinivas NR, Barr WH, Shyu WC, et al. Bioequivalence of two tablet formulations of nadolol using single and multiple dose data: assessment using stereospecific and nonstereospecific assays. *J Pharm Sci*. 1996;85(3):299-303. doi:10.1021/js950442m

144. Misaka S, Miyazaki N, Yatabe MS, et al. Pharmacokinetic and pharmacodynamic interaction of nadolol with itraconazole, rifampicin and grapefruit juice in healthy volunteers. *J Clin Pharmacol*. 2013;53(7):738-745. doi:10.1002/jcph.95

145. Krukemyer JJ, Boudoulas H, Binkley PF, Lima JJ. Comparison of single-dose and steady-state nadolol plasma concentrations. *Pharm Res*. 1990;7(9):953-956.

146. Morganroth J, Duchin KL. Effectiveness of low-dose nadolol for ventricular arrhythmias. *Am J Cardiol*. 1986;58(3):273-278.

147. Kamali F, Howes A, Thomas SH, Ford GA, Snoeck E. A pharmacokinetic and pharmacodynamic interaction study between nebivolol and the H2-receptor antagonists cimetidine and ranitidine. *Br J Clin Pharmacol*. 1997;43(2):201-204.

148. Briciu C, Neag M, Muntean D, et al. A pharmacokinetic drug interaction study between nebivolol and paroxetine in healthy volunteers. *J Clin Pharm Ther*. 2014;39(5):535-540. doi:10.1111/jcpt.12180

149. Luo X, Lei Y, He L, et al. No influence of CYP2D6*10 genotype and phenotype on the pharmacokinetics of nebivolol in healthy Chinese subjects. *J Clin Pharm Ther*. 2015;40(5):561-565. doi:10.1111/jcpt.12310

150. Briciu C, Neag M, Muntean D, et al. Phenotypic differences in nebivolol metabolism and bioavailability in healthy volunteers. *Clujul Med 1957*. 2015;88(2):208-213. doi:10.15386/cjmed-395

151. Neves DV, Lanchote VL, Moysés Neto M, Cardeal da Costa JA, Vieira CP, Coelho EB. Influence of chronic kidney disease and haemodialysis treatment on pharmacokinetics of nebivolol enantiomers. *Br J Clin Pharmacol*. 2016;82(1):83-91. doi:10.1111/bcp.12917

152. Gheldiu A-M, Popa A, Neag M, et al. Assessment of a Potential Pharmacokinetic Interaction between Nebivolol and Bupropion in Healthy Volunteers. *Pharmacology*. 2016;98(3-4):190-198. doi:10.1159/000447266

153. Vespasiano CFP, Laurito TL, Iwamoto RD, Moreno RA, Mendes GD, De Nucci G. Bioequivalence study between a fixed-dose single-pill formulation of nebivolol plus hydrochlorothiazide and separate formulations in healthy subjects using high-performance liquid chromatography coupled to tandem mass spectrometry. *Biomed Chromatogr BMC*. 2017;31(5). doi:10.1002/bmc.3884

154. Gheldiu A-M, Vlase L, Popa A, et al. Investigation of a Potential Pharmacokinetic Interaction Between Nebivolol and Fluvoxamine in Healthy Volunteers. *J Pharm Pharm Sci Publ Can Soc Pharm Sci Soc Can Sci Pharm*. 2017;20:68-80.

155. Chen CL, Desai-Krieger D, Ortiz S, Kerolous M, Wright HM, Ghahramani P. A Single-Center, Open-Label, 3-Way Crossover Trial to Determine the Pharmacokinetic and Pharmacodynamic Interaction Between Nebivolol and Valsartan in Healthy Volunteers at Steady State. *Am J Ther*. 2015;22(5):e130-140. doi:10.1097/MJT.0000000000000247

156. Kendall MJ. Pharmacokinetics of oxprenolol in the elderly. *Am J Cardiol*. 1983;52(9):54D-56D.

157. Bradbrook ID, John VA, Morrison PJ, Rogers HJ, Spector RG. Pharmacokinetic investigation of the absorption of oxprenolol from Oros delivery systems in healthy volunteers: comparison of in vivo and in vitro drug release. *Br J Clin Pharmacol*. 1985;19 Suppl 2:163S-169S.

158. Gupta PK, Lim JK, Zoest AR, Lam FC, Hung CT. Relative bioavailability of oral sustained-release and regular-release oxprenolol tablets at steady-state. *Biopharm Drug Dispos*. 1991;12(7):493-503.

159. Gugler R, Kreis L, Dengler HJ. Pharmacokinetics of a new beta-adrenoceptor blocking agent, LF 17-895, in man. *Arzneimittelforschung*. 1975;25(7):1067-1072.

160. Jennings GL, Bobik A, Fagan ET, Korner PI. Pindolol pharmacokinetics in relation to time course of inhibition of exercise tachycardia. *Br J Clin Pharmacol*. 1979;7(3):245-256.

161. Aellig WH, Nüesch E, Pacha W. Pharmacokinetic comparison of pindolol 30 mg retard and 15 mg normal tablets. *Eur J Clin Pharmacol*. 1982;21(6):451-455.

162. Juma FD. Pharmacokinetics of pindolol in Kenyan Africans. *Eur J Clin Pharmacol*. 1983;25(3):425-426.

163. Krause W, Lennert C. Pharmacokinetics of mepindolol administered alone and in combination with hydrochlorothiazide--a bioequivalence study. *Biopharm Drug Dispos*. 1983;4(4):339-345.

164. Somogyi AA, Bochner F, Sallustio BC. Stereoselective inhibition of pindolol renal clearance by cimetidine in humans. *Clin Pharmacol Ther*. 1992;51(4):379-387.

165. Koch HJ, Raschka C, Hannak D. Bioequivalence of two oral immediate release formulations of pindolol in healthy volunteers assessed by ratio analysis. *Acta Physiol Pharmacol Bulg*. 2000;25(3-4):99-102.

166. Hitzenberger G, Fitscha P, Beveridge T, Nüesch E, Pacha W. Effects of age and smoking on the pharmacokinetics of pindolol and propranolol. *Br J Clin Pharmacol*. 1982;13(Suppl 2):217S-222S.

167. Holmes D, Nuesch E, Houle JM, Rosenthaler J. Steady state pharmacokinetics of hydrolysed bopindolol in young and elderly men. *Eur J Clin Pharmacol*. 1991;41(2):175-178. doi:10.1007/BF00265913

168. Gugler R, Bodem G. Single and multiple dose pharmacokinetics of pindolol. *Eur J Clin Pharmacol*. 1978;13(1):13-16.

169. Bianchetti G, Graziani G, Brancaccio D, et al. Pharmacokinetics and effects of propranolol in terminal uraemic patients and in patients undergoing regular dialysis treatment. *Clin Pharmacokinet*. 1976;1(5):373-384.

170. Heagerty AM, Donovan MA, Castleden CM, Pohl JF, Patel L, Hedges A. Influence of cimetidine on pharmacokinetics of propranolol. *Br Med J Clin Res Ed*. 1981;282(6280):1917-1919.

171. Perucca E, Grimaldi R, Gatti G, et al. Pharmacokinetic and pharmacodynamic studies with a new controlled-release formulation of propranolol in normal volunteers: a comparison with other commercially available formulations. *Br J Clin Pharmacol*. 1984;18(1):37-43.

172. Williams FM, Leeser JE, Rawlins MD. Pharmacodynamics and pharmacokinetics of single doses of ketanserin and propranolol alone and in combination in healthy volunteers. *Br J Clin Pharmacol*. 1986;22(3):301-308.

173. Straka RJ, Lalonde RL, Pieper JA, Bottorff MB, Mirvis DM. Nonlinear pharmacokinetics of unbound propranolol after oral administration. *J Pharm Sci*. 1987;76(7):521-524.

174. Biswas NR, Garg SK, Lal R, et al. Pharmacokinetic study of a new sustained release preparation of propranolol in normal healthy volunteers. *Int J Clin Pharmacol*. 1988;26(9):436-438.

175. Flouvat B, Berlin I, Cournot A, et al. Pharmacokinetic and pharmacodynamic comparison of two doses of long acting propranolol (80 and 160 mg) in healthy subjects. *Br J Clin Pharmacol*. 1989;27(5):539-545.

176. Walle T, Walle UK, Cowart TD, Conradi EC. Pathway-selective sex differences in the metabolic clearance of propranolol in human subjects. *Clin Pharmacol Ther*. 1989;46(3):257-263.

177. Schoors DF, Vercruysse I, Musch G, Massart DL, Dupont AG. Influence of nicardipine on the pharmacokinetics and pharmacodynamics of propranolol in healthy volunteers. *Br J Clin Pharmacol*. 1990;29(5):497-501.

178. Zhou HH, Wood AJ. Differences in stereoselective disposition of propranolol do not explain sensitivity differences between white and Chinese subjects: correlation between the clearance of (-)- and (+)-propranolol. *Clin Pharmacol Ther*. 1990;47(6):719-723.

179. Bano G, Raina RK, Zutshi U, Bedi KL, Johri RK, Sharma SC. Effect of piperine on bioavailability and pharmacokinetics of propranolol and theophylline in healthy volunteers. *Eur J Clin Pharmacol*. 1991;41(6):615-617. doi:10.1007/BF00314996

180. Bennett PN, Fenn GC, Notarianni LJ, Lee CE. Misoprostol does not alter the pharmacokinetics of propranolol. *Postgrad Med J*. 1991;67(787):455-457.

181. Bleske BE, Welage LS, Rose S, Amidon GL, Shea MJ. The effect of dosage release formulations on the pharmacokinetics of propranolol stereoisomers in humans. *J Clin Pharmacol*. 1995;35(4):374-378.

182. Power JM, Morgan DJ, McLean AJ. Effects of sensory (teasing) exposure to food on oral propranolol bioavailability. *Biopharm Drug Dispos*. 1995;16(7):579-589.

183. Xie HG, Chen X. Sex differences in pharmacokinetics of oral propranolol in healthy Chinese volunteers. *Zhongguo Yao Li Xue Bao*. 1995;16(5):468-470.

184. Buch A, Barr WH. Absorption of propranolol in humans following oral, jejunal, and ileal administration. *Pharm Res*. 1998;15(6):953-957.

185. Mansur AP, Avakian SD, Paula RS, Donzella H, Santos SR, Ramires JA. Pharmacokinetics and pharmacodynamics of propranolol in hypertensive patients after sublingual administration: systemic availability. *Braz J Med Biol Res Rev Bras Pesqui Médicas E Biológicas Soc Bras Biofísica Al*. 1998;31(5):691-696.

186. Wójcicki J, Sulzyc-Bielicka V, Kutrzeba J, Gawrońska-Szklarz B, Droździk M, Sterna Z. Studies on the pharmacokinetics and pharmacodynamics of propranolol in hyperlipidemia. *J Clin Pharmacol*. 1999;39(8):826-833.

187. Wójcicki J, Jaroszynska M, Droździk M, Pawlik A, Gawrońska-Szklarz B, Sterna R. Comparative pharmacokinetics and pharmacodynamics of propranolol and atenolol in normolipaemic and hyperlipidaemic obese subjects. *Biopharm Drug Dispos*. 2003;24(5):211-218. doi:10.1002/bdd.357

188. Sica D, Frishman WH, Manowitz N. Pharmacokinetics of propranolol after single and multiple dosing with sustained release propranolol or propranolol CR (innopran XL) , a new chronotherapeutic formulation. *Heart Dis Hagerstown Md*. 2003;5(3):176-181. doi:10.1097/01.HDX.0000074436.09658.3b

189. Mehuys E, Remon JP, Korst A, et al. Human bioavailability of propranolol from a matrix-in-cylinder system with a HPMC-Gelucire core. *J Control Release Off J Control Release Soc*. 2005;107(3):523-536. doi:10.1016/j.jconrel.2005.06.019

190. Salman S a. B, Amrah S, Wahab MSA, et al. Modification of propranolol’s bioavailability by Eurycoma longifolia water-based extract. *J Clin Pharm Ther*. 2010;35(6):691-696. doi:10.1111/j.1365-2710.2009.01147.x

191. Salehifar E, Ebrahim S, Shiran M-R, et al. Pharmacokinetic Parameters and Over-Responsiveness of Iranian Population to Propranolol. *Adv Pharm Bull*. 2017;7(2):195-202. doi:10.15171/apb.2017.024

192. Walle T, Fagan TC, Conradi EC, Walle UK, Gaffney TE. Presystemic and systemic glucuronidation of propranolol. *Clin Pharmacol Ther*. 1979;26(2):167-172.

193. Wilson TW, Firor WB, Johnson GE, et al. Timolol and propranolol: bioavailability, plasma concentrations, and beta blockade. *Clin Pharmacol Ther*. 1982;32(6):676-685.

194. Walle T, Walle UK, Olanoff LS, Conradi EC. Partial metabolic clearances as determinants of the oral bioavailability of propranolol. *Br J Clin Pharmacol*. 1986;22(3):317-323.

195. Lalonde RL, Pieper JA, Straka RJ, Bottorff MB, Mirvis DM. Propranolol pharmacokinetics and pharmacodynamics after single doses and at steady-state. *Eur J Clin Pharmacol*. 1987;33(3):315-318.

196. Roberts RJ, Leff RD. Influence of absorbable and nonabsorbable lipids and lipidlike substances on drug bioavailability. *Clin Pharmacol Ther*. 1989;45(3):299-304.

197. Lalonde RL, Bottorff MB, Straka RJ, Tenero DM, Pieper JA, Wainer IW. Nonlinear accumulation of propranolol enantiomers. *Br J Clin Pharmacol*. 1988;26(1):100-102.

198. Zhou HH, Whelan E, Wood AJ. Lack of effect of ageing on the stereochemical disposition of propranolol. *Br J Clin Pharmacol*. 1992;33(1):121-123.

199. Reimann IW, Klotz U, Siems B, Frölich J. Cimetidine increases steady state plasma levels of propranolol. *Br J Clin Pharmacol*. 1981;12(6):785-790.

200. Dvornik D, Kraml M, Dubuc J, Patterson-Kreuscher S, Milosovich G, Mullane JF. Comparative bioavailability of propranolol: twice-daily versus four times-daily administration. *J Clin Pharmacol*. 1981;21(11-12 Pt 1):472-476.

201. Garg DC, Jallad NS, Mishriki A, et al. Comparative pharmacodynamics and pharmacokinetics of conventional and long-acting propranolol. *J Clin Pharmacol*. 1987;27(5):390-396.

202. Henry D, Brent P, Whyte I, Mihaly G, Devenish-Meares S. Propranolol steady-state pharmacokinetics are unaltered by omeprazole. *Eur J Clin Pharmacol*. 1987;33(4):369-373.

203. Sharoky M, Perkal M, Turner R, Lesko LJ. Steady state relative bioavailability and pharmacokinetics of oral propranolol in black and white North Americans. *Biopharm Drug Dispos*. 1988;9(5):447-456.

204. Kowey PR, Kirsten EB, Fu CH, Mason WD. Interaction between propranolol and propafenone in healthy volunteers. *J Clin Pharmacol*. 1989;29(6):512-517.

205. Dimmitt DC, Yu DK, Elvin AT, Giesing DH, Lanman RC. Pharmacokinetics of diltiazem and propranolol when administered alone and in combination. *Biopharm Drug Dispos*. 1991;12(7):515-523.

206. Murdoch DL, Thomson GD, Thompson GG, Murray GD, Brodie MJ, McInnes GT. Evaluation of potential pharmacodynamic and pharmacokinetic interactions between verapamil and propranolol in normal subjects. *Br J Clin Pharmacol*. 1991;31(3):323-332.

207. Stoschitzky K, Lindner W, Egginger G, et al. Racemic (R,S)-propranolol versus half-dosed optically pure (S)-propranolol in humans at steady state: Hemodynamic effects, plasma concentrations, and influence on thyroid hormone levels. *Clin Pharmacol Ther*. 1992;51(4):445-453.

208. Shaw-Stiffel TA, Walker SE, Ogilvie RI, Leenen FH. Pharmacokinetic and pharmacodynamic interactions during multiple-dose administration of nisoldipine and propranolol. *Clin Pharmacol Ther*. 1994;55(6):661-669.

209. Marathe PH, Greene DS, Kollia GD, Barbhaiya RH. A pharmacokinetic interaction study of avitriptan and propranolol. *Clin Pharmacol Ther*. 1998;63(3):367-378. doi:10.1016/S0009-9236(98)90168-0

210. Leahey WJ, Neill JD, Varma MP, Shanks RG. Comparison of the efficacy and pharmacokinetics of conventional propranolol and a long acting preparation of propranolol. *Br J Clin Pharmacol*. 1980;9(1):33-40.

211. McAinsh J, Holmes BF, Baber NS, Young J. Bioavailability of propranolol and bendrofluazide formulations. *Biopharm Drug Dispos*. 1981;2(2):167-175.

212. Bennett PN, Fenn GC, Notarianni LJ. Potential drug interactions with misoprostol: effects on the pharmacokinetics of antipyrine and propranolol. *Postgrad Med J*. 1988;64 Suppl 1:21-24.

213. Eldon MA, Kinkel AW, Daniel JE, Latts JR. Bioavailability of propranolol hydrochloride tablet formulations: application of multiple dose crossover studies. *Biopharm Drug Dispos*. 1989;10(1):69-76.

214. Zhou HH, Koshakji RP, Silberstein DJ, Wilkinson GR, Wood AJ. Altered sensitivity to and clearance of propranolol in men of Chinese descent as compared with American whites. *N Engl J Med*. 1989;320(9):565-570. doi:10.1056/NEJM198903023200905

215. Poirier JM, Jaillon P, Lecocq B, Lecocq V, Ferry A, Cheymol G. The pharmacokinetics of d-sotalol and d,l-sotalol in healthy volunteers. *Eur J Clin Pharmacol*. 1990;38(6):579-582.

216. Le Coz F, Funck-Brentano C, Poirier JM, Kibleur Y, Mazoit FX, Jaillon P. Prediction of sotalol-induced maximum steady-state QTc prolongation from single-dose administration in healthy volunteers. *Clin Pharmacol Ther*. 1992;52(4):417-426.

217. Uematsu T, Kanamaru M, Nakashima M. Comparative pharmacokinetic and pharmacodynamic properties of oral and intravenous (+)-sotalol in healthy volunteers. *J Pharm Pharmacol*. 1994;46(7):600-605.

218. Kimura M, Umemura K, Ikeda Y, et al. Pharmacokinetics and pharmacodynamics of (+/-)-sotalol in healthy male volunteers. *Br J Clin Pharmacol*. 1996;42(5):583-588.

219. Somberg JC, Preston RA, Ranade V, Molnar J. QT prolongation and serum sotalol concentration are highly correlated following intravenous and oral sotalol. *Cardiology*. 2010;116(3):219-225. doi:10.1159/000316050

220. Darpo B, Karnad DR, Badilini F, et al. Are women more susceptible than men to drug-induced QT prolongation? Concentration-QTc modelling in a phase 1 study with oral rac-sotalol. *Br J Clin Pharmacol*. 2014;77(3):522-531. doi:10.1111/bcp.12201

221. Barbey JT, Sale ME, Woosley RL, Shi J, Melikian AP, Hinderling PH. Pharmacokinetic, pharmacodynamic, and safety evaluation of an accelerated dose titration regimen of sotalol in healthy middle-aged subjects. *Clin Pharmacol Ther*. 1999;66(1):91-99. doi:10.1016/S0009-9236(99)70058-5

222. Fiset C, Philippon F, Gilbert M, Turgeon J. Stereoselective disposition of (+/-)-sotalol at steady-state conditions. *Br J Clin Pharmacol*. 1993;36(1):75-77.

223. Lowenthal DT, Pitone JM, Affrime MB, et al. Timolol kinetics in chronic renal insufficiency. *Clin Pharmacol Ther*. 1978;23(5):606-615.

224. Mäntylä R, Männistö P, Nykänen S, Koponen A, Lamminsivu U. Pharmacokinetic interactions of timolol with vasodilating drugs, food and phenobarbitone in healthy human volunteers. *Eur J Clin Pharmacol*. 1983;24(2):227-230.

225. McGourty JC, Silas JH, Fleming JJ, McBurney A, Ward JW. Pharmacokinetics and beta-blocking effects of timolol in poor and extensive metabolizers of debrisoquin. *Clin Pharmacol Ther*. 1985;38(4):409-413.
